# Supplementary material for: Microbiome-pathogen interactions drive epidemiological dynamics of antibiotic resistance: A modeling study applied to nosocomial pathogen control
Source: eLife. 2021 Sep 14;10:e68764. doi: 10.7554/eLife.68764 (PMC8560094; doi:10.7554/eLife.68764)
Supplement: Supplementary file 1. [file elife-68764-supp1.docx]

**EXPERT ELICITATION: Within-host interactions between bacteria, and consequences for antibiotic stewardship**

David SMITH^1^, Laura TEMIME, Lulla OPATOWSKI
1. david.smith@pasteur.fr

EXPERT: ____________________________ DATE: ____________________________

**OBJECTIVES:**

You have been invited to participate in an **expert elicitation** exercise. Expert elicitation is a scientific consensus methodology, which involves the formal **estimation of parameter values and their uncertainty from subject-matter experts.**

Using this document and with David’s assistance, you will be asked to quantify your belief in the values of **epidemiological parameters** related to (i) commensal flora, (ii) high-risk multidrug-resistant bacteria (MRB), (iii) interactions between them, and (iv) consequences for antibiotic use.

The parameter values we gather will be used to inform a **mathematical model** that describes the impact of **within-host MRB-flora interactions** on the transmission dynamics of MRB in the healthcare setting, in the context of four high-risk species: *Clostridioides difficile*, methicillin-resistant *Staphylococcus aureus*, ESBL-producing *Escherichia coli*, and carbapenemase-producing *Klebsiella pneumoniae.*

While completing this exercise, remember that:

- **There is no single correct answer** to any question you will be asked
- Your answers will never be taken on their own, but will be **combined** with those of other experts (n>10) into final “pooled” distributions
- Your answers will be treated as **confidential** **and** **anonymous**
- You will not be held personally accountable for this work

If you so wish, we will be pleased to thank you in the acknowledgements section of any publications resulting from this work.

Would you like to be acknowledged by name for your inclusion in this work? ________

**SCIENTIFIC CONTEXT:**

Host microbiota (‘flora’) live in diverse polymicrobial communities that normally exist at **stable equilibrium** over the course of years to decades. **Within-host interactions** between bacteria affect the ability of many species to colonize their hosts (1). Interactions include competition for resources, metabolite cross-feeding, biofilm formation and many more (2).

**Antibiotic** use can result in large-scale disruption of host flora, entailing **(i)** reduced abundance and diversity of commensal microbes, **(ii)** increased susceptibility to MRB colonization, and **(iii)** selection for antibiotic resistance genes (Fig. 1) (3,4,5). This antibiotic-disrupted state is referred to as **flora dysbiosis** and varies depending on the antibiotic used (Fig. 2).

Restricting antibiotic use through **antibiotic stewardship interventions** is proposed to benefit public health not only by reducing selection for pre-existing MRB, but also by preventing disruption of commensal – and often protective – flora.


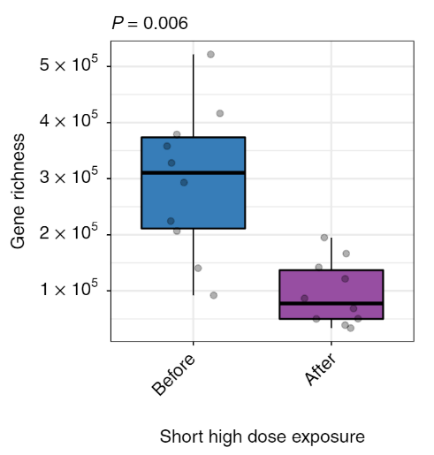

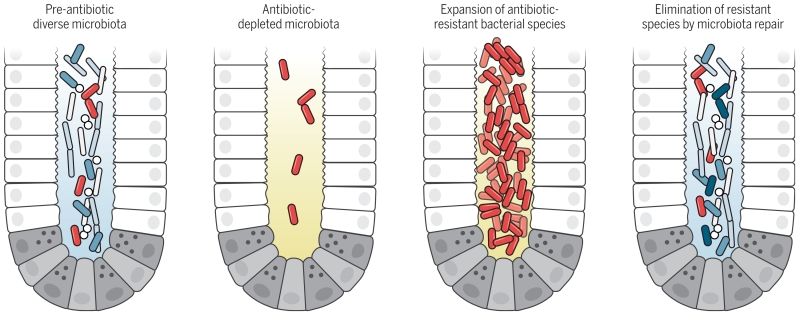


**Figure 1: Left:** Illustration of how antibiotics disrupt digestive microbiota (6). **Right:** Microbiota gene richness in patients before and after selective digestive decontamination with broad-spectrum antibiotics (3)


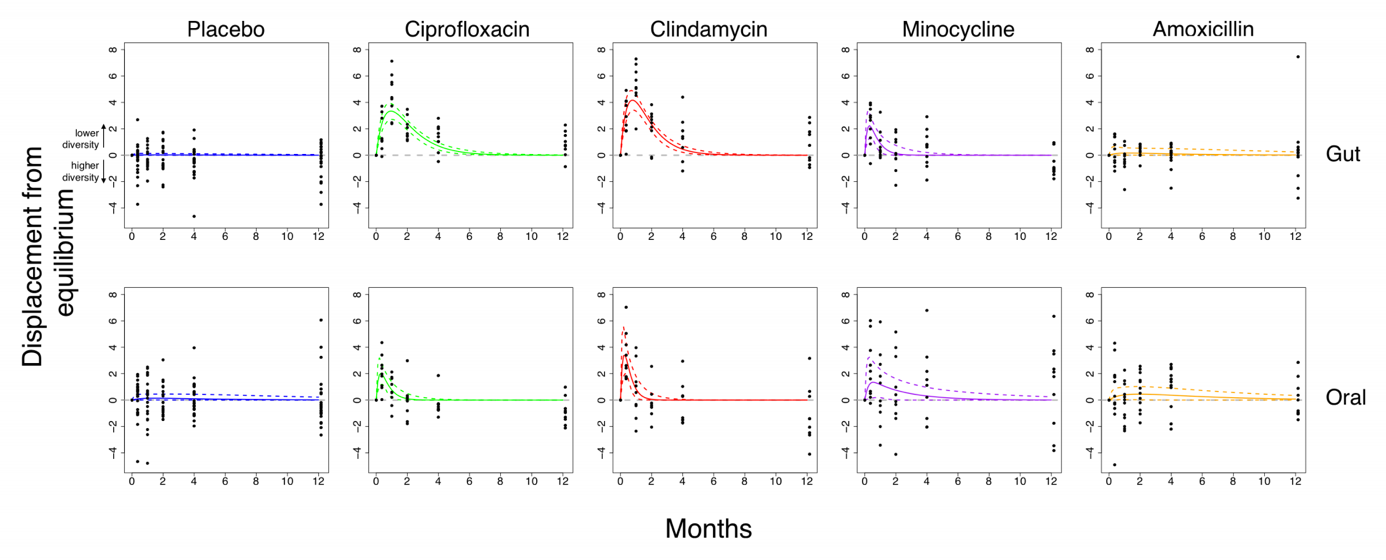


**Figure 2.** Model estimates of changes in microbial diversity subsequent to antibiotic use. Y-axis = displacement from equilibrium; x-axis = time (months) (7)

**EXPERT ELICITATION**

Throughout this exercise you will be asked to quantify your beliefs and uncertainty in the numerical values of a range of different parameters. David will walk you through how this works, using the MATCH expert elicitation tool (Fig. 3) (<http://optics.eee.nottingham.ac.uk/match/uncertainty.php>).


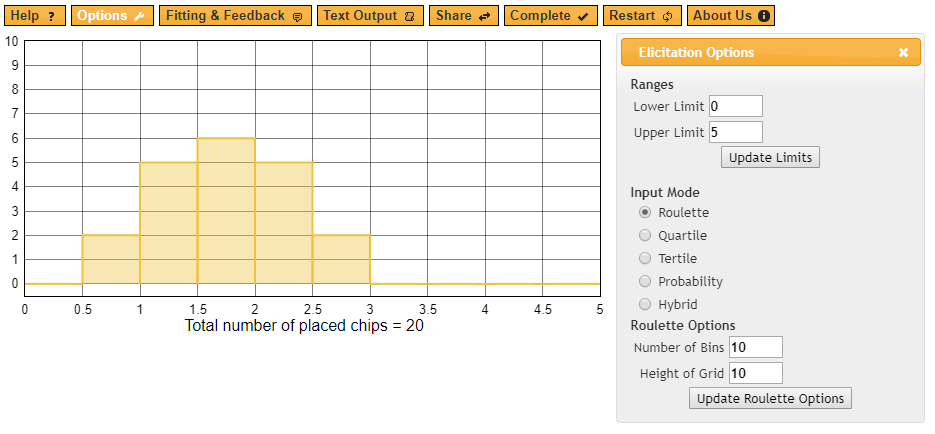


**Figure 3.** An illustration of using “chips and bins” to build histograms demonstrating your belief and uncertainty in a parameter value.

**Practice Question:**

| **QUESTION** | **DISTRIBUTION** |
| --- | --- |
| What proportion of patients in my hospital undergo antibiotic therapy at any given time? |  |

**Biases to consider:**

1. **Availability bias:** consider all relevant information when answering, and not only information that you have heard most recently.
2. **Representativeness bias:** recognize the specificity of what you know, and avoid over-generalizing.
3. **Over-confidence bias:** consider the full range of all possible values for each parameter.
4. **Motivational bias:** try not to let moral or professional obligations sway your thinking.
5. **Anchoring bias:** try not to let an early estimate bias or “anchor” later estimates.

**Tips for making estimates:**

1. **Consider the ‘best’ answer first and extremes second.** Consider building distributions by starting with the mean, followed by the highest and lowest possible values.
2. **Think out loud.** Reasoning orally can help you to locate information.
3. **Trust your gut.**
4. **Let David know if you would like to ‘qualify’ any of your answers**: if you wish, you can provide additional information not captured by a particular question.

**PART I. Interactions between strains**

In some bacterial species, colonization with one serotype or “strain” can inhibit colonization with another (e.g. as in pneumococcus; illustrated in Fig. 4). However, in other species, diverse strains may overlap within the host without competitively excluding one another. In other species still, only a very few different strains circulate at any given time.

**Figure 4.** Illustration of epidemiological strain competition.

| **QUESTION** | **SPECIES** | **YES/NO/PARTIALLY** |
| --- | --- | --- |
| Does **“strain competition”** affect the epidemiology of this species in hospital patients?  In other words, does colonization with certain strains or serotypes of this species (e.g., a drug-sensitive strain) **prevent colonization** with a different strain or serotype (e.g., a drug-resistant strain)? | *C. difficile* | (1a) |
|  | *S. aureus* | (1b) |
|  | *E. coli* | (1c) |
|  | *K. pneumoniae* | (1d) |

**PART II. Interactions between species**

We make three key assumptions about how MRB and flora interact:

1. A bacteria’s **vital epidemiological parameters** (acquisition, colonization duration, growth) are affected by interactions with host flora (Table 2).
2. Interactions vary depending on whether or not patients have normal **“equilibrium flora**” or antibiotic-disrupted “**dysbiotic flora**” (Fig. 5).
3. MRB can be **sub-dominant** (low density, negligible epidemiologic transmission) or can “grow out” and become **dominant** (greater density, transmissible).

**Table 2.** Within-host mechanisms of interaction between commensal flora and MRB.

|  | **Bacterial epidemiology** | **Interaction with flora** | **Example** |
| --- | --- | --- | --- |
| **1** | **Acquisition** | Flora prevent MRB from establishing new colonies (‘colonization resistance’) | Bifidobacteria bacteriocins inhibit colonization with *C. difficile* and *E. coli* (8) |
| **2** | **Colonization duration** | Flora compete with MRB for space and resources | Bacteroides bacteriocins reduce colonization duration in *E. faecalis*, *Listeria* (9) |
| **3** | **Within-host growth** | Loss of flora enhances growth of sub-dominant MRB | *C. scindens* bile acids prevent outgrowth of subdominant *C. difficile* colonies (5) |
| **4** | **Horizontal gene transfer** | MRB and flora exchange antibiotic resistance genes | Exchange of plasmids bearing CTX-M-15 ESBL genes, particularly in *E. coli* ST131 and *K. pneumoniae* ST258 (10) |

| **Equilibrium flora** | **Dysbiotic flora** |
| --- | --- |
| 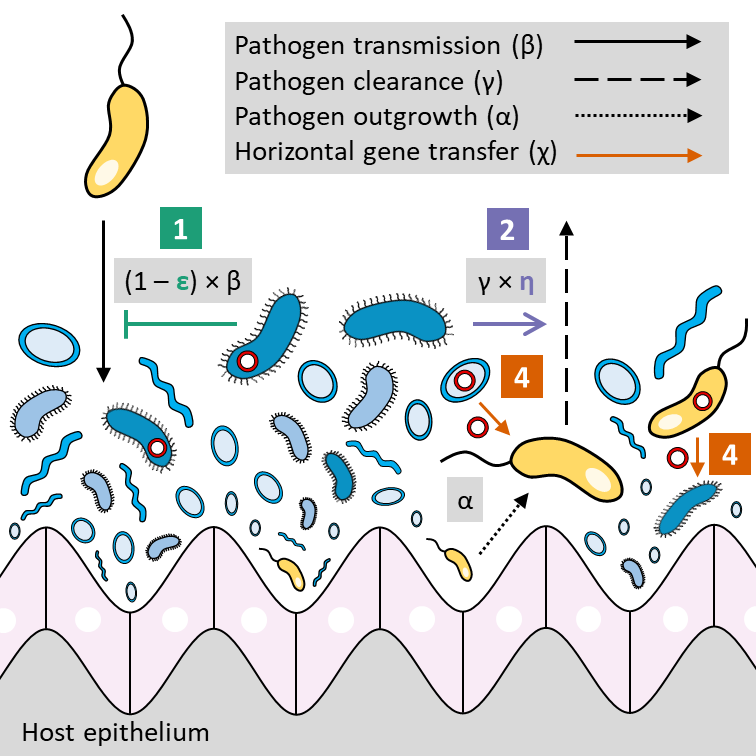 | 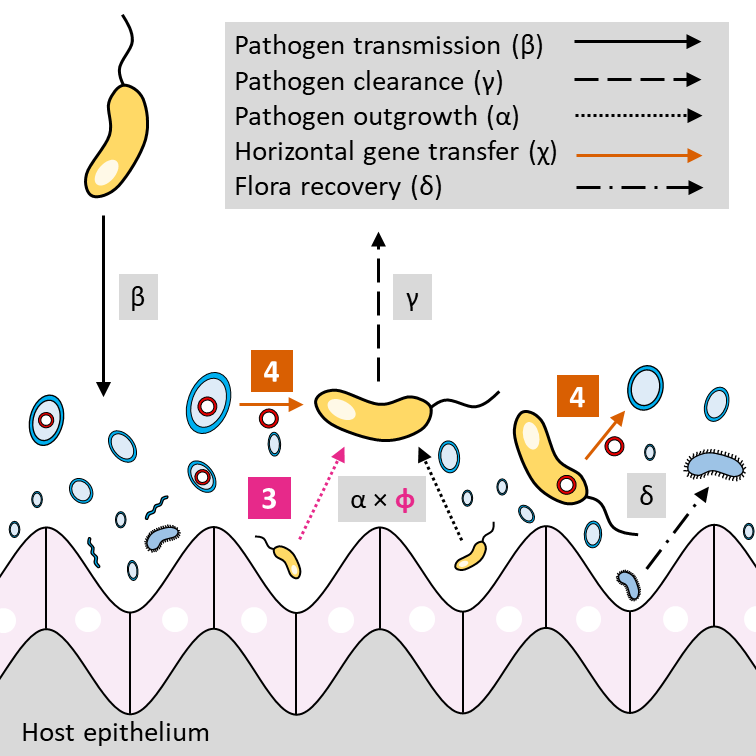 |
| **LEGEND**  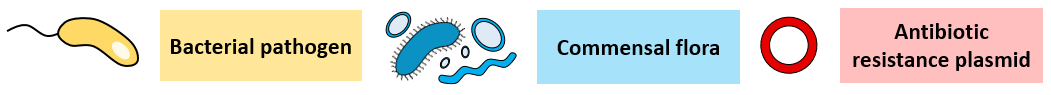 | |

**Figure 5.** Differences in within-host flora-MRB interactions in hosts with normal healthy flora (left) and with dysbiotic flora (right).

In the following questions, you will be asked to estimate the **relative risk** of epidemiological events in patients with dysbiotic flora as compared to patients with normal equilibrium flora.


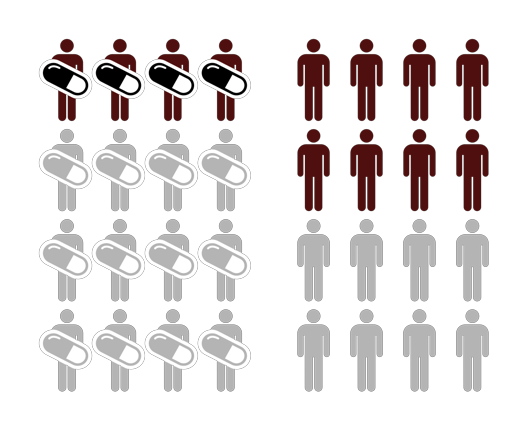
Remember that **relative risk (RR)** refers to the ratio of the probability of an outcome in group B compared to the reference group A (Figure 6).

**Figure 6.** For a given disease outcome (**brown**), patients not on medication (**right, 8/16**) are twice as affected as patients on medication (**left, 4/16**). Therefore, the **relative risk (RR)** of this outcome in non-treated patients is **8/4=2**.

For each question, please consider bacterial **colonization** (not necessarily infection) in a “normal” patient population in the hospital setting in which you work most closely. Remember that most MRB rarely cause disease: asymptomatic colonization is the rule, opportunistic infection is the exception.

**Mechanism 1: Acquisition**

| **QUESTION** | **SPECIES** | **YES/NO** | **IF YES…** | **DISTRIBUTION** |
| --- | --- | --- | --- | --- |
| Do patients with dysbiotic flora **acquire** this bacteria more easily than patients with equilibrium flora? | *C. difficile* |  | Compared to patients with equilibrium flora, **what is the relative risk of acquisition** in patients with dysbiotic flora (**RR**)? | (2a) |
|  | *MRSA* |  |  | (2b) |
|  | *ESBL-E. coli* |  |  | (2c) |
|  | *CP-K. pneumoniae* |  |  | (2d) |

**Mechanism 2: Colonization duration**

| **QUESTION** | **SPECIES** | **YES/NO** | **IF YES…** | **DISTRIBUTION** |
| --- | --- | --- | --- | --- |
| Does bacterial colonization **persist** for a longer duration in patients with dysbiotic flora compared to patients with equilibrium flora? | *C. difficile* |  | **How much longer is the duration of colonization** in patients with dysbiotic flora compared to patients with equilibrium flora (in **days**)? | (3a) |
|  | *MRSA* |  |  | (3b) |
|  | *ESBL-E. coli* |  |  | (3c) |
|  | *CP-K. pneumoniae* |  |  | (3d) |

**Mechanism 3: Growth**

| **QUESTION** | **SPECIES** | **YES/NO** | **IF YES…** | **DISTRIBUTION** |
| --- | --- | --- | --- | --- |
| Do you believe that **subdominant** bacterial populations are more likely to **“grow out”** (become dominant) in patients with dysbiotic flora than those with equilibrium flora? | *C. difficile* |  | Compared to patients with equilibrium flora, what is the **relative risk** **of a subdominant population**  **growing out** (becoming dominant) in patients with dysbiotic flora (**RR**)? | (4a) |
|  | *MRSA* |  |  | (4b) |
|  | *ESBL-E. coli* |  |  | (4c) |
|  | *CP-K. pneumoniae* |  |  | (4d) |

**Mechanism 4: Horizontal gene transfer**

| **QUESTION** | **SPECIES** | **YES/NO/VERY RARELY** | **IF YES…** | **DISTRIBUTION** |
| --- | --- | --- | --- | --- |
| Does this species acquire antibiotic resistance genes horizontally from digestive flora (e.g. via plasmids)? | *C. difficile* |  | Among patients colonized with this bacteria while taking antibiotics, what **proportion of patients** will acquire **new resistance via horizontal gene transfer** over the course of their hospital stay (**%**)? | (5a) |
|  | *S. aureus* |  |  | (5b) |
|  | *E. coli* |  |  | (5c) |
|  | *K. pneumoniae* |  |  | (5d) |

| **QUESTION** | **SPECIES** | **YES/NO** | **IF YES…** | **DISTRIBUTION** |
| --- | --- | --- | --- | --- |
| Assuming resistance genes are present among patient microbiota, does horizontal gene transfer occur more frequently among patients with dysbiotic flora compared to patients with equilibrium flora? | *C. difficile* |  | Compared to patients with equilibrium flora, what is the **relative risk** **of acquiring new resistance via horizontal gene transfer** in patients with dysbiotic flora (**RR**)? | (6a) |
|  | *S. aureus* |  |  | (6b) |
|  | *E. coli* |  |  | (6c) |
|  | *K. pneumoniae* |  |  | (6d) |

| **QUESTION** | **NAME and/or CONTACT DETAILS** |
| --- | --- |
| Can you share the name and/or contact details for any other subject-matter experts who may be interested in participating in this exercise? |  |

| **QUESTION** | **COMMENTS** |
| --- | --- |
| Do you have any comments you wish to formally share for consideration in interpretation or analysis of your estimates?  Included in this, **do you have any financial or professional conflicts of interest to declare?** |  |

**Thank you !**

Thank you very kindly for your participation. Please don’t hesitate to be in touch if you have any questions or comments about this exercise.

**Bibliography**

1. Bäumler AJ, Sperandio V. Interactions between the microbiota and pathogenic bacteria in the gut. Nature. 2016 Jul 7;535(7610):85–93.

2. Kamada N, Chen GY, Inohara N, Núñez G. Control of pathogens and pathobionts by the gut microbiota. Nat Immunol. 2013 Jul;14(7):685–690.

3. Ruppé E, Ghozlane A, Tap J, Pons N, Alvarez A-S, Maziers N, et al. Prediction of the intestinal resistome by a three-dimensional structure-based method. Nat Microbiol. 2019;4(1):112–123.

4. Thiébaut ACM, Arlet G, Andremont A, Papy E, Sollet J-P, Bernède-Bauduin C, et al. Variability of intestinal colonization with third-generation cephalosporin-resistant Enterobacteriaceae and antibiotic use in intensive care units. J Antimicrob Chemother. 2012 Jun;67(6):1525–1536.

5. Hajishengallis G, Lamont RJ. Dancing with the Stars: How Choreographed Bacterial Interactions Dictate Nososymbiocity and Give Rise to Keystone Pathogens, Accessory Pathogens, and Pathobionts. Trends Microbiol. 2016 Mar 8;24(6):477–489.

6. Pamer EG. Resurrecting the intestinal microbiota to combat antibiotic-resistant pathogens. Science. 2016 Apr 29;352(6285):535–538.

7. Shaw LP, Bassam H, Barnes CP, Walker AS, Klein N, Balloux F. Modelling microbiome recovery after antibiotics using a stability landscape framework. ISME J. 2019 Mar 15;13(7):1845–1856.

8. Buffie CG, Pamer EG. Microbiota-mediated colonization resistance against intestinal pathogens. Nat Rev Immunol. 2013 Nov;13(11):790–801.

9. Hooper LV, Stappenbeck TS, Hong CV, Gordon JI. Angiogenins: a new class of microbicidal proteins involved in innate immunity. Nat Immunol. 2003 Mar;4(3):269–273.

10. Mathers AJ, Peirano G, Pitout JDD. The role of epidemic resistance plasmids and international high-risk clones in the spread of multidrug-resistant Enterobacteriaceae. Clin Microbiol Rev. 2015 Jul;28(3):565–591.
